# Precision Temperature Control System with Low EMI for Applications in Analyzing Thermal Properties of Highly Sensitive Piezoelectric Sensors

**DOI:** 10.3390/s22218525

**Published:** 2022-11-05

**Authors:** Sylwester Nowocień, Radosław Sławomir Wielgus, Janusz Mroczka

**Affiliations:** Department of Electronic and Photonic Metrology, Wroclaw University of Science and Technology, 50-317 Wroclaw, Poland

**Keywords:** precision thermal stabilization, quartz crystal microbalance (QCM), thermoelectric cooler (TEC), resonant frequency, Peltier module driver, QCM signal quality

## Abstract

A low electromagnetic interference (EMI), precision temperature control system for sensitive piezoelectric sensors stabilization and their thermal characteristics research was proposed. Quartz crystal microbalance (QCM) was chosen as the device to be tested. Recently, QCMs found use in many fields of study such as biology, chemistry, and aerospace. They often operate in harsh environments and are exposed to many external factors including temperature fluctuations, to which QCMs are highly susceptible. Such disturbances can cause undesirable resonant frequency shifts resulting in measurement errors that are difficult to eliminate. The proposed solution enables measurements of QCMs thermal characteristics, effectiveness evaluation of temperature compensation methods, and testing of the frequency stability. As a part of the developed solution, two independent temperature regulators were used: first to maintain the QCM crystal at desired temperature, and second to keep the QCM oscillator circuit at fixed temperature. The single regulator consists of a thermoelectric module (TEC) used for both heating and cooling. Two considered TEC driving methods were compared in terms of EMI and their impact on the QCM signal quality. The proposed system was examined for its temperature stabilization capability showing high stability of 11 mK_p-p_ for one hour and the setpoint accuracy of ±15 mK in the full temperature range.

## 1. Introduction

Temperature changes are one of the main sources of uncertainties in many physical measurement systems [1,2]. Thermal effects such as stress and strain leading to displacement, bending, and deformation of materials can impair system measuring capabilities by changing parameters of its mechanical parts [3]. These effects are especially important for precision piezoelectric sensors, such as QCM, considering its principle of operation relies on mechanical resonance. It consists of thin piece of quartz with metallization on opposing sides providing electrical contacts. When alternating voltage is applied to electrodes, the quartz changes its shape due to the piezoelectric effect. As a result, microbalance starts to resonate at the certain frequency which can be controlled by its mechanical dimensions. The QCM operation principle is like that of crystal oscillators used for the time and frequency control, but the QCM surface is exposed to the surrounding environment. This allows for external factors to impact the resonant frequency and damping factor of the exposed crystal, which makes it possible to indirectly measure various physical quantities. QCM properties make it suitable for many applications in modern science such as biosensors [4,5,6,7], vapors and particulate matter sensors [8,9,10], systems for characterization of liquids [11,12,13], and aerospace [14,15,16,17,18]. Underlying most experiments are indirect measurements of mass changes and viscoelastic properties [19]. Changes in the QCM resonant frequency due to the mass adsorption can be described by the Sauerbrey equation [20]:

(1) $$\Delta f=-\frac{2f_{0}^{2}}{A\sqrt{\rho_{q}\mu_{q}}}\Delta m$$

where: Δf—frequency change, Δm—mass change, $f_{0}$—fundamental resonant quartz frequency, A—active crystal area, $\rho_{q}$—quartz density, and $\mu_{q}$—quartz shear modulus.

Similarly, the change in the QCM resonant frequency due to the liquid loading can be calculated using the Kanazawa–Gordon equation [21]:

(2) $$\Delta f=-f_{0}^{\frac{2}{3}}\sqrt{\frac{\rho_{l}\eta_{l}}{\pi G_{q}\rho_{q}}}$$

where: Δf—frequency change, $f_{0}$—fundamental resonant quartz frequency, $\rho_{q}$—quartz density, $\mu_{q}$—quartz shear modulus, $\rho_{l}$—liquid density, and $\eta_{l}$—liquid viscosity. Equations (1) and (2) are widely used for measurements in soft matter research [19]. There is a lot of recent research in this field in which QCMs are used in wide temperature ranges. One example is the listeria monocytogenes detection method developed by Wachiralurpan, S. et al. In this study, QCM was used as the DNA hybridization biosensor operating at 60 °C [6]. Another example is research on low-temperature adsorption and fouling of milk fractions on stainless steel conducted by Holly A. Huellemeier et al. in 25, 50, and 65 °C [22]. In work of Nicoletta Braidotti et al., viscoelastic properties of rat cardiac fibroblasts cytoskeletons were investigated after the treatment with cytochalasin D and nocodazole using QCM kept in 37 °C [23]. Another common use of QCM is the thin-film deposition monitoring. In this case, QCMs are often used in extreme conditions, especially in case of aerospace applications. In Kazutaka Nishiyama and Hitoshi Kuninaka’s paper, QCM for the contamination monitoring outside of the SDS-4 satellite was developed. Shortly after launch, the frequency increase was observed likely caused by the QCM surface erosion, then frequency started to decrease indicating the contaminants’ deposition [15]. In this application, QCM was exposed to temperatures ranging from −40 to 80 °C. In another aerospace-related publication by Yuta Tsuchiya et al., QCM for materials outgassing and elemental oxygen detection in vacuum was described. In this case, an even higher operating temperature range of −190 to 125 °C was considered [16]. It is not difficult to see that the lack of consideration of all thermal effects during such experiments could lead to significant increase of measurement uncertainties. These phenomena are very important, especially since changes in the frequency due to temperature changes can be on the comparable level or even higher than those caused by measured physical phenomena.

**Figure 1:**
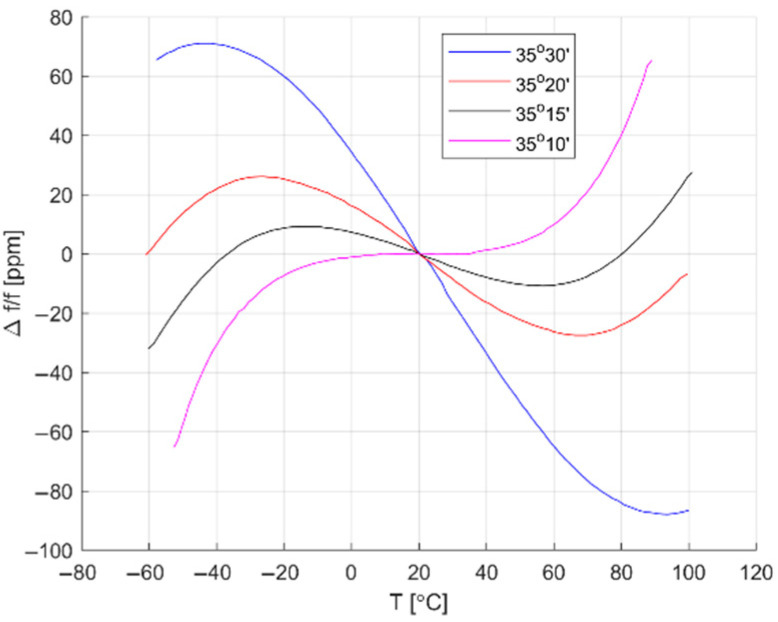
AT-cut quartz crystal thermal characteristics for different cut angles [24].

Similar to crystal oscillators, the most popular QCM type is AT-cut, hence thermal characteristics are similar. Resonant frequency temperature dependence of AT-cut crystal oscillator is shown in Figure 1.

As one can see, temperature changes can severely impact measurements results. For example, for 35°30′ AT-cut QCM with 5 MHz fundamental frequency and the round electrode with diameter of 5 mm operating at 60 °C the frequency will change by −64.8 ppm. This is equal to a 324 Hz frequency drop, which according to Equation (1) corresponds to the 1.12 µg mass increase at the QCM surface, which has to be compensated. In Wachiralurpan, S. et al. paper measurements were also performed at 60 °C [6]; therefore temperature effects have to be compensated but also the temperature stability has to be taken into account. According to the user manual of Thermo Haake^®^ DC30, the thermostat used during discussed experiments has a temperature stability of ±10 mK and accuracy of ±100 mK, which for measured mass change of 300 ng adds up to uncertainty of ±4.95 ng. These considerations show that temperature changes can severely impact measurements results and the precise temperature control is essential for high accuracy measurements, QCM characterization, and verification of temperature compensation methods [13,17].

In many integrated measurement systems, resistive heaters or TECs are placed close to QCM for the effective temperature control. However, in this configuration, QCM is exposed to local electromagnetic interference emissions from the thermal actuator [9,10,16,17,18,25]. In some cases, PWM or other type of high frequency square wave signal is utilized for the thermal actuator power control [10,25]. It is a well-known fact that such signals introduce EMI to the environment [26,27,28], but a thermal actuator driving method and its impact on measurement results is not always considered. An innovative aspect of following work is the demonstration and analysis of how the QCM signal quality is impacted by an interference introduced by certain thermal actuator control methods, thus degrading measurement accuracy. A novel, low EMI thermal control system utilizing developed TEC controller was proposed.

## 2. Materials and Methods

In order to determine the TEC control method effect on the QCM signal quality, a controlled environment was needed. For this reason, in the first phase of work, a thermally insulated test chamber with a control and measurement system was developed to achieve this goal.

### 2.1. Mechanical Setup

**Figure 2:**
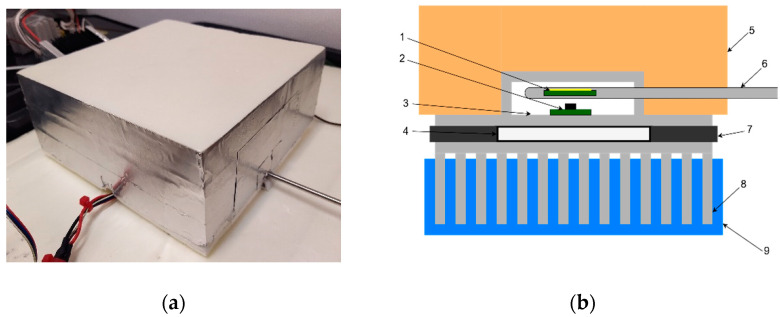
Proposed test chamber (**a**) assembled unit; (**b**) schematic diagram (1−QCM assembly, 2−temperature sensor, 3−aluminum plate, 4−TEC, 5−extruded polystyrene insulation, 6−Testo Pt100 probe, 7−thermal insulation, 8−heatsink, 9−water bath).As presented in Figure 2b, TEC (4) was placed between an aluminum plate (3) and heatsink (8). The plate serves as the thermally stabilized working surface. The precision temperature sensor (Texas Instruments TMP116) (2) for PI loop controller was mounted on the plate top using TermoPasty AG TermoGlue. The heatsink was submerged in a 30 L water bath (9) allowing for the heat dissipation necessary to achieve negative temperatures at the working surface. A centrifugal pump was used to create a constant water flow through heatsink for the heated water exchange. High temperature differences exist between the working surface and heatsink at maximum and minimum temperature setpoints, hence, an insulating layer of felt (7) was placed around TEC to reduce the heat flow between its sides which increased insulation. An aluminum chamber was placed on the top of the working surface creating enclosed air volume. TermoPasty AG Silver thermal paste was applied on the area of contact between the chamber and the working surface. The chamber core was surrounded by 60 mm of extruded polystyrene insulation (5) to reduce the energy exchange with environment. The air temperature inside the chamber was measured using Testo high-precision immersion/penetration Pt100 probe (6). The developed QCM assembly (1) was suspended in the middle of the test chamber. It consists of 14 mm 5 MHz AT-cut QCM with round electrode with diameter of 5 mm from Quartz pro, Sweden, mounted on PCB quartz crystal holder as presented in Figure 3.

Firstly, the test chamber was designed and assembled. A photo of the finished device is shown in Figure 2a.

The PCB has a hole with diameter slightly smaller than QCM allowing for adhesion of overlapping surfaces at the quartz crystal edge. Electrodes were soldered to pads on the PCB surface.

### 2.2. Electronics

In the next phase of work, the chamber control system was developed. Its structure is shown in Figure 4.

MCU handles temperature measurements, reads the setpoint provided by a PC user, sends the measured temperature to PC for logging purposes, and calculates the PI controller output value. The calculated output is translated to voltage generated by a digital to analog converter used to control TEC driver output. The microcontroller utilizes error which is defined as the difference between the setpoint provided by the user and the temperature measured at working surface. The controller transfer function is expressed with Equation (3):

(3) $$G\left(s\right)=K_{p}+\frac{K_{i}}{s}$$

where $K_{p}$ and $K_{i}$ are the proportional and integral gains [29]. To prevent integral overflow, dynamic integral clamping was used. It prevents the controller from integrating when output is already saturated by the proportional gain output. Integrator limits $I_{max}$ and $I_{min}$ are calculated using Equations (4) and (5).

(4) $$I_{max}=\max\left\{y_{max}-P,\;0\right\}$$

(5) $$I_{min}=\min\left\{y_{min}-P,\;0\right\}$$

where $y_{max}$ and $y_{min}$ are PI controller output limits and P is the proportional gain output. As temperature changes are relatively slow, sampling time was not critical, $T_{s}=200$ ms was chosen. The controller was tuned using the manual method.

### 2.3. System Calibration and Adjustment

Before measurements with QCM were taken, temperature at the working surface was calibrated and adjusted. The Testo Pt100 probe (3) was placed directly on the surface next to the temperature sensor (1) as presented in Figure 5.

The TermoPasty AG Silver thermal paste was applied on probe tip (2) for the better thermal coupling. The setpoint was changed from −3 °C to 70 °C with 5 °C step. For every setpoint value, when the temperature was within ±10 mK for at least 10 min, the temperature was measured with a Testo 735-2 thermometer. Differences between setpoint and obtained data samples were calculated giving the setpoint error before the adjustment as shown in Figure 6. Error is the resultant of the temperature sensor accuracy and indirect coupling between the temperature sensor and working surface.

To reduce the setpoint error, linear regression method was used. The error before adjustment was approximated with the line equation which was implemented in the MCU allowing for the temperature measurements correction. The calibration process was repeated to determine the maximum error in the full temperature range. A result of ±15 mK was achieved.

### 2.4. Measurement Setup

The next step was preparation of the test bench for precision frequency measurements, allowing for the QCM oscillator circuit output signal analysis. The measurement setup diagram is presented in Figure 7.

The Testo 735-2 thermometer was connected to PC with Testo ComSoft v4.5 software used for the data logging. QCM was driven by the series-resonance two-inverter oscillator circuit as it does not require external capacitances parallel to the quartz crystal, thus reducing number of temperature sensitive components which could introduce additional frequency error during measurements [30]. An oscillator circuit was fitted with its own independent temperature stabilization system keeping it at constant 25 °C. Tektronix MSO5204 oscilloscope with DpoJet application was used for frequency measurements and the time interval error (TIE) analysis. IQOV-164-4 OCXO was used as the reference frequency source for oscilloscope time base. Every sample was calculated as mean of 10,000 frequency values calculated using single signal periods. Frequency measurements were taken when temperature was within ±10 mK for at least 10 min. Using the developed test, bench experiments were carried out to determine the impact of TEC driving methods on the QCM output signal quality.

### 2.5. Pulse Width Modulation Driving Method

Firstly, a 50 kHz PWM signal for precise TEC power control was used. TIE degradation was observed when the temperature stabilization was enabled. To obtain TIE, the real QCM signal was compared with the expected signal as shown in Figure 8a. Expected signal frequency was equal to the mean frequency calculated from 10,000 real signal periods. Time differences between the occurrences of expected and real signal edges were measured for all periods as shown in Figure 8b.

The TIE of the QCM output signal was measured in two configurations: without any thermal stabilization as reference and then with the temperature stabilization control enabled. The histograms of obtained TIE values are compared in Figure 9a.

**Figure 3:**
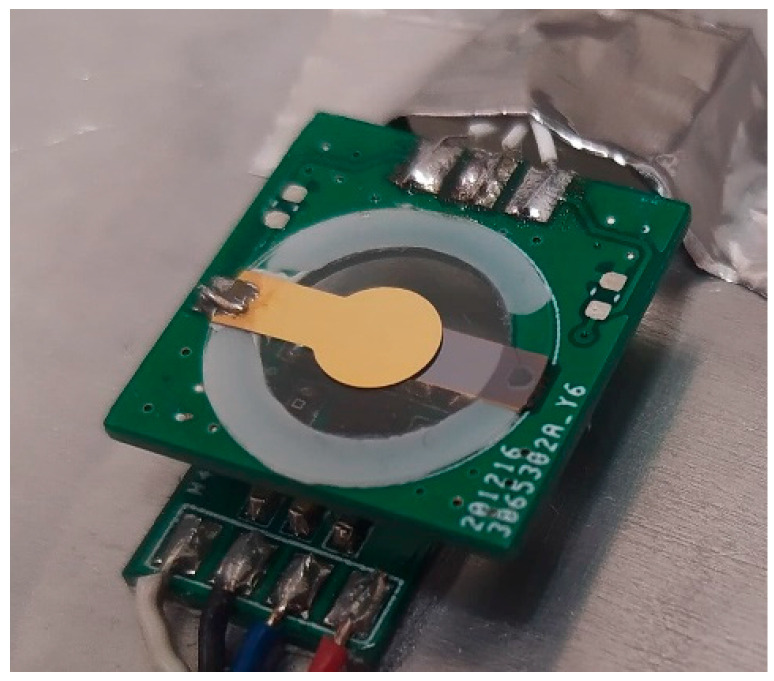
QCM mounted on proposed custom PCB holder.

**Figure 4:**
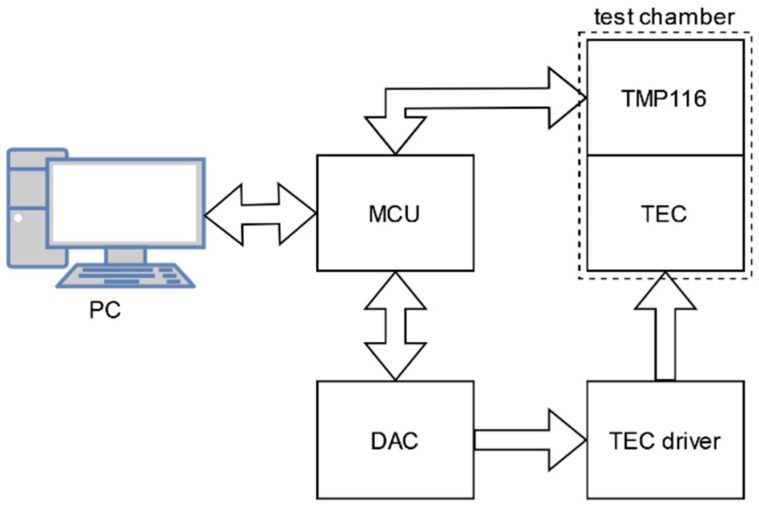
The chamber control system—block diagram.

**Figure 5:**
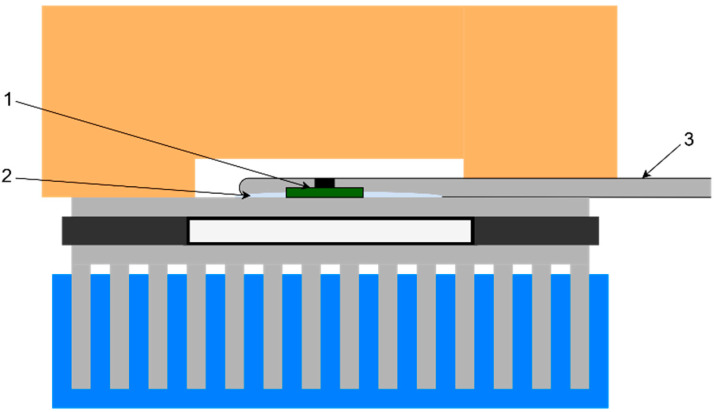
Test chamber setup for surface temperature calibration (1−temperature sensor, 2−thermal paste, 3−Testo Pt100 probe).

**Figure 6:**
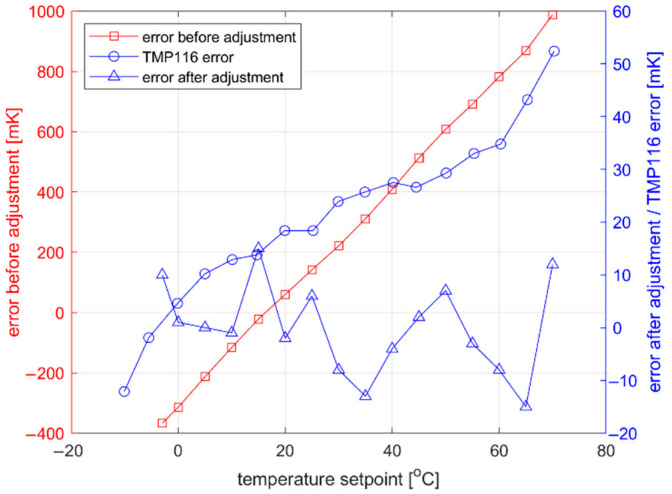
Setpoint error of the proposed system before and after calibration.

**Figure 7:**
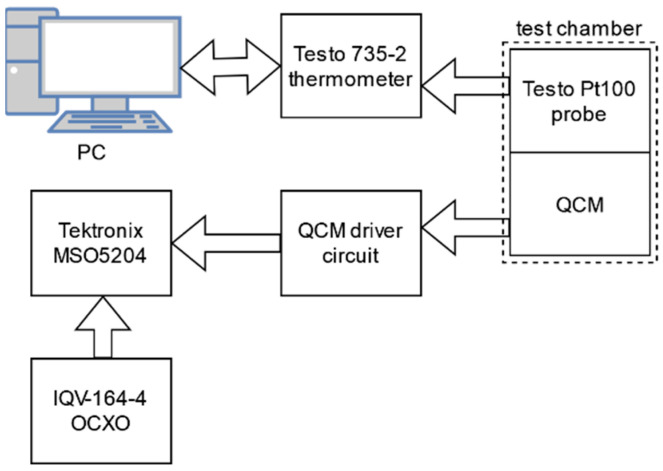
Measurement setup block diagram.

**Figure 8:**
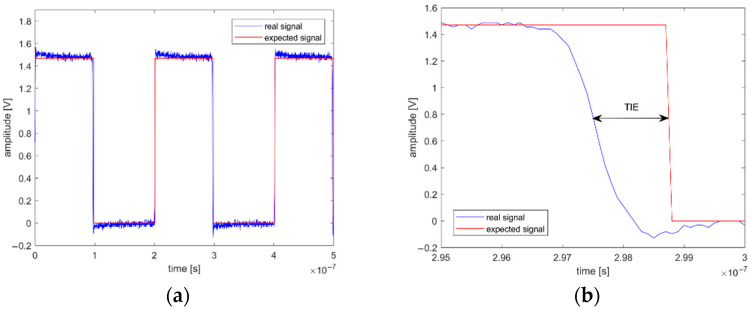
Comparison of raw QCM signal (**a**) real and expected periods; (**b**) real and expected edges.

**Figure 9:**
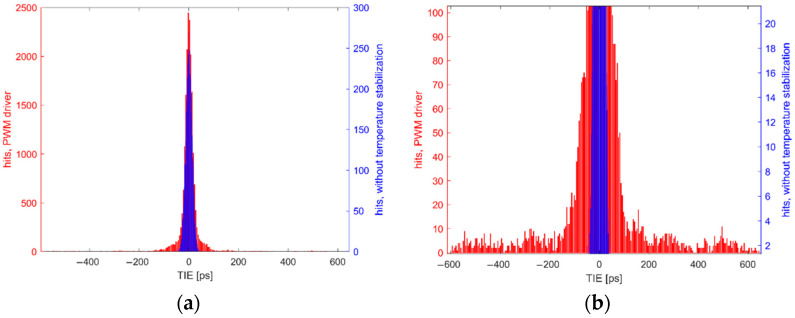
Distribution of TIE values (**a**) with and without temperature stabilization; (**b**) closeup on histograms tails.

**Figure 10:**
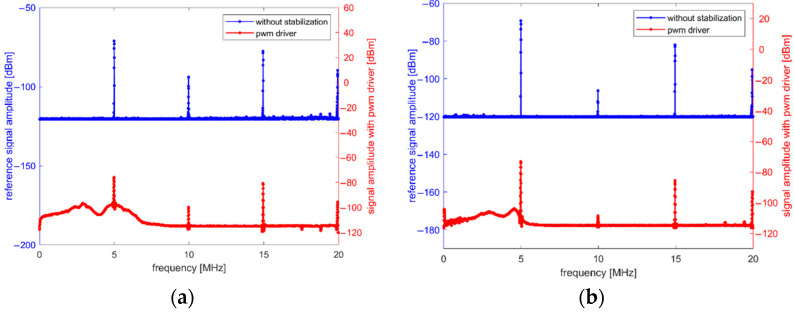
Frequency spectrums of reference QCM signal and QCM signal with PWM driver enabled (**a**) electric field; (**b**) magnetic field.

The TIE of the oscillator output signal without thermal stabilization shown in Figure 9 has the standard deviation of 13.118 ps and with PWM signal activated, this value increased to 76.854 ps, which indicates that oscillator had become much more unstable. Peak TIE value reached 644.2 ps, hence the histogram for measurement taken with PWM driver enabled is heavy tailed as shown in Figure 3b. This value is equal to a frequency change of 160,533 Hz and according to Equation (1), corresponds to a mass change of 55.7 µg. Such instabilities in QCM signal can result in high measurement errors, especially for short gating times. To confirm that PWM-induced EMI is source of the TIE degradation, measurements of electric and magnetic fields were performed 10 mm above the QCM surface with near-field ProbeSet model PBS1 and RTSA BB60C spectrum analyzer. This method was chosen because it allows to locate the source of interference close to the device under test and to measure the magnitude of the fields directly affecting the QCM signal quality [31,32], which was critical for the conducted research. For each field type, a reference measurement was taken first without the temperature stabilization and then a second measurement was taken with PWM driver enabled. QCM oscillator output signal is a square wave; therefore, fundamental quartz frequency and its harmonics can be seen on the graphs. The frequency spectra of electric and magnetic fields are presented in Figure 10a,b, respectively.

In Figure 10, when PWM driver is enabled, wideband noise is present in signal spectrum even despite grounded shielding for both driver and TEC. Hence, another driver solution should be considered when the high accuracy of QCM measurements is required.

### 2.6. Bipolar Constant Current Source

**Figure 11:**
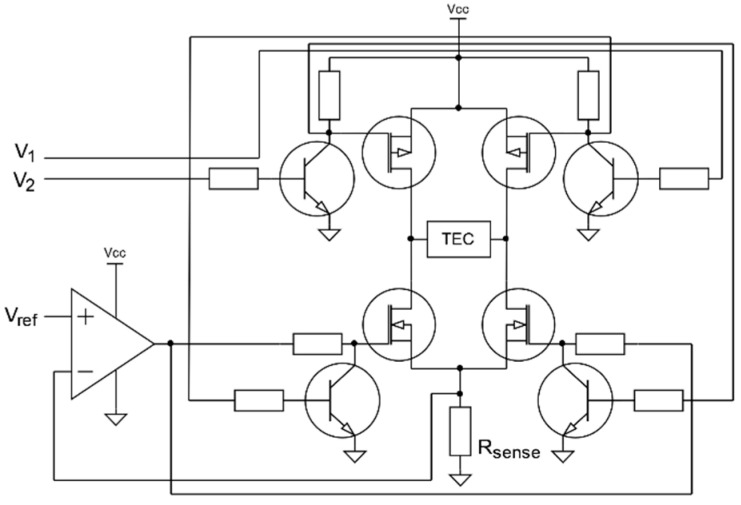
Simplified schematic of the proposed bipolar current source.

To avoid high frequency switching noise observed in measurements with PWM TEC driver, bipolar constant current source was developed for the TEC control. Its simplified schematic diagram is presented in Figure 11.

The proposed solution uses modified H-bridge. High side P-channel MOSFETs are used as simple switches with NPN bipolar transistors used for voltage level translation. Low side N-channel MOSFETs in combination with an op-amp serve as voltage controlled current sources. Each MOSFET can be disabled by shorting gate with ground trough NPN bipolar transistor. The current source is controlled by three signals. *V*_1_ and *V*_2_ are logic level voltages used to control current flow direction. The value of current flowing through the TEC module is controlled by $V_{ref}$ voltage generated by the digital to analog converter. Op-amp compares reference voltage $V_{ref}$ with voltage drop on sensing resistor $R_{sense}$ and drives currently enabled N-channel MOSFET to reduce difference between these two values. The presented solution allows to control TEC power precisely with a single power supply, which is a clear advantage.

## 3. Results

In order to verify the proposed system performance in terms of EMI, temperature accuracy, and stability, a series of measurements and analyses were carried out, the results of which are presented below.

### 3.1. EMI

**Figure 12:**
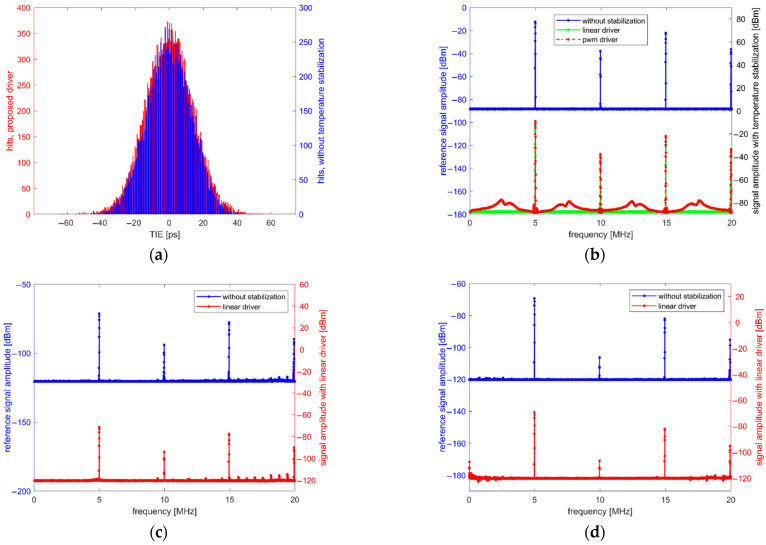
Measurement results with the proposed TEC controller. (**a**) TIE distribution of direct oscillator output; (**b**) direct oscillator output frequency spectrum; (**c**) electric field frequency spectrum; (**d**) magnetic field frequency spectrum.

To determine how the proposed TEC driver impacts the QCM signal quality and to compare its performance to the PWM driver, measurements from Section 2.5 were repeated. Results were shown in Figure 12.

The results of measurements of the proposed system clearly show much better performance. The reference TIE histogram is now comparable to the histogram obtained with the temperature stabilization enabled as shown in Figure 12a. The value of standard deviation of the TIE histogram measured with temperature stabilization enabled is equal to 14.097 ps, which is much closer to the value for measurement performed without the temperature stabilization (13.118 ps). Such results indicate that the proposed solution is superior to the PWM driver in terms of interference introduced to a raw output signal. This is additionally confirmed by the frequency spectrum of raw oscillator output signal presented in Figure 12b. No significant difference can be seen between the signal without temperature stabilization and with the linear driver enabled as opposed to measurement with the PWM driver enabled. Similarly, the measurements of electric and magnetic fields in Figure 12c,d show that the interference introduced by proposed solution is negligible.

### 3.2. Long-Term Stability

**Figure 13:**
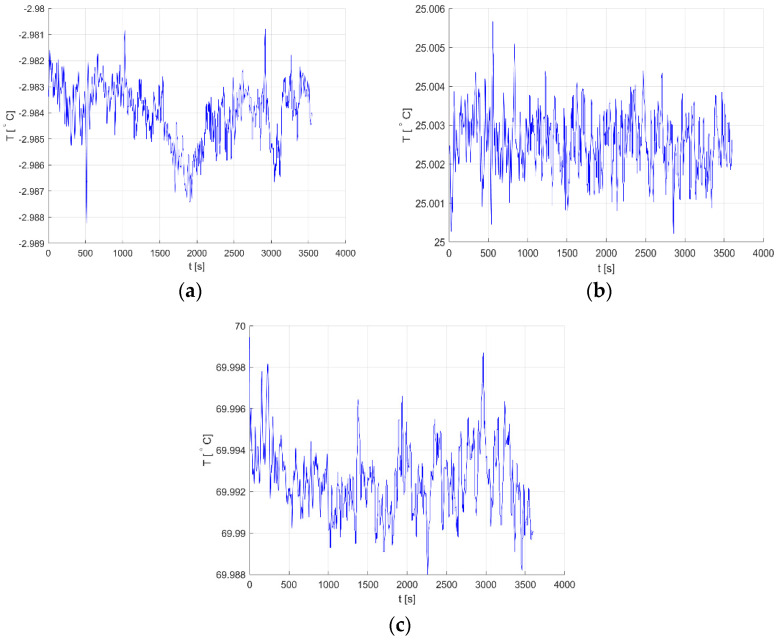
One-hour temperature stability of proposed system for three setpoints (**a**) −3 °C; (**b**) 25 °C; (**c**) 70 °C.

QCM-based measurements often take tens of minutes to complete [4,6], hence the proposed system was tested for the long-term stability. Three setpoint values were chosen: 25 °C which is very often used for electronic components testing and characterization, and highest and lowest stable temperatures achieved, i.e., −3 °C and 70 °C. Results are shown in Figure 13.

For a −3 °C setpoint temperature, fluctuations do not exceed 8mK_p-p_ (Figure 9a); for 25 °C, they are below 6mK_p-p_ (Figure 9b); and for 70 °C, they are within a range of 11mK_p-p_ (Figure 9c). If an achieved result of 6mK_p-p_ would be considered for 35°30′ AT-cut quartz crystal from Section 1 operating at 25 °C, this would correspond to 0.005Hz_p-p_ frequency fluctuation or 17.3pg_p-p_ mass fluctuation.

## 4. Conclusions

A temperature control system suitable for sensitive piezoelectric sensors (e.g., QCM) was developed and its performance was examined. Thanks to the use of the TEC module and the linear bipolar current source, the device is relatively simple, has no moving parts, and offers a low level of EMI which was experimentally confirmed. Results indicate that an inappropriate TEC driving method can disturb the raw sensor signal and introduce more noise to measurement results than temperature changes. As has been shown in Section 2.3, a setpoint accuracy of ±15 mK was achieved in the full temperature range after the calibration and adjustment of the proposed system. One-hour temperature stability was also evaluated, maximum fluctuations of 11 mK_p-p_ were measured. A comparison of obtained results with the commercial solution analyzed in the first section shows that the developed system provides several times better temperature stability, which proves its ability to expand the applicability of discussed sensors. A deeper analysis of the proposed solution and its application for practical issues will be subject of future development research in the field of improving QCM-based measurement techniques.

## Data Availability

The data presented in this study are available on request from the corresponding author.
